# Mitral Annular Disjunction: Epidemiology, Diagnostic Methods, Prognosis, and Novel Implications

**DOI:** 10.3390/jcdd12080311

**Published:** 2025-08-18

**Authors:** Vasileios Tsimpiris, Georgia Kousourna, Aristi Boulmpou, Magdalini Petridou, Chalil Tsavousoglou, Dimitrios Kotzadamis, Christodoulos Papadopoulos, Dimitrios Ntelios, Theodoros Moysiadis, Vassilios Vassilikos, Efstathios Pagourelias

**Affiliations:** 1 Third Department of Cardiology, Aristotle University of Thessaloniki, Ippokratio General Hospital, PC 54642 Thessaloniki, Greece; tsimpiris@gapps.auth.gr (V.T.); aristi_bou1993@yahoo.gr (A.B.); vvassil@auth.gr (V.V.); 2 Department of Computer Science, School of Sciences and Engineering, University of Nicosia, 2417 Nicosia, Cyprus; moysiadis.t@unic.ac.cy

**Keywords:** mitral annular disjunction, mitral valve prolapse, cardiac arrhythmias, sudden cardiac death

## Abstract

Mitral annular disjunction (MAD) is an increasingly recognized structural abnormality of the mitral valve apparatus, often associated with mitral valve prolapse and a heightened risk of ventricular arrhythmias and sudden cardiac death. It is defined by a separation between the mitral annulus and the left ventricular myocardium, best visualized during systole. In this review, we present an updated and comprehensive overview of MAD, drawing from recent large-scale imaging studies, expert consensus documents, and newly proposed classifications such as true versus pseudo-MAD. We discuss its prevalence, anatomical features, and diagnostic challenges across multiple imaging modalities, including transthoracic and transesophageal echocardiography, cardiovascular magnetic resonance, and computed tomography. We also explore its pathophysiological role in arrhythmogenesis, its prognostic implications, and current management strategies. Special attention is given to risk stratification based on imaging and cardiac rhythm findings, and we propose a practical clinical framework to guide decision-making. This review aims to support clinicians in recognizing MAD as a potentially arrhythmogenic condition that requires systematic evaluation and follow-up.

## 1. Introduction

Mitral annular disjunction (MAD) is a structural abnormality of the mitral valve apparatus that has recently been a subject of scientific interest. First described over 30 years ago, it involves a separation between the mitral valve annulus and the basal portion of the left ventricle, typically observed during systole. Although historically associated with mitral valve prolapse (MVP), recent research suggests that MAD may represent a distinct entity with important implications for cardiac function and arrhythmogenesis [1].

MAD has been increasingly recognized as a potential contributor to both mechanical dysfunction and electrical instability, raising concerns about its role in adverse cardiac outcomes, including ventricular arrhythmias and sudden cardiac death (SCD) [2]. Advances in imaging modalities, such as transthoracic echocardiography (TTE), transesophageal echocardiography (TTE), and cardiovascular magnetic resonance (CMR), have greatly enhanced our ability to identify and characterize this condition, while also revealing variations in its prevalence and clinical manifestations [3,4].

Despite these advancements, the pathophysiology of MAD remains incompletely understood; whether it is a congenital anomaly or an acquired condition secondary to structural and degenerative processes is still under investigation [5]. Furthermore, its close association with MVP, myxomatous mitral valve degeneration, and connective tissue disorders highlights the complexity and heterogeneity of this condition [6].

In this review, we aim to provide a comprehensive overview of MAD, focusing on its epidemiology, diagnostic techniques, prognostic significance, and emerging therapeutic considerations. By synthesizing the existing literature, we hope to offer valuable insights into this evolving field and its implications for clinical practice.

## 2. Epidemiology

The absence of a standardized definition for MAD combined with variations in imaging techniques and differences in its recognition by physicians and sonographers have resulted in inconsistencies in the reported prevalence within the general population. According to recent reports, the prevalence of MAD ranges between 7.2% and 8.7% [3]. A large autopsy study in a Chinese population analyzing 1373 dissected hearts found a notably high prevalence of MAD, namely 92.1% across the entire mitral annulus and 74.9% specifically at the posterior annulus, suggesting that MAD may be a far more common anatomical finding than previously thought [7]. Evidence suggests a higher incidence in females and a notable prevalence in younger age groups [8,9]. In the same context, other studies have demonstrated a strong association between female gender and the development of MAD when considering alongside factors such as age, left ventricular ejection fraction (LVEF), left ventricular end-diastolic diameter, and the severity of mitral regurgitation [10,11].

The relationship between MAD and MVP is of particular interest, as the two frequently coexist [6]. Among patients with MAD, the prevalence of MVP has been reported to be 78%; conversely, MAD has been identified in 20–58% of patients with MVP [12]. A recent meta-analysis of 23 imaging studies comprising 7718 MVP patients reported a pooled MAD prevalence of approximately 40%, although estimates varied widely between 5.4% and 90% depending on the diagnostic modality and criteria used [8]. Specifically, the prevalence of MAD was 47% using CMR, 44.7% with TEE, 31.3% with TTE, and 20% with cardiac computed tomography (CT). These findings underscore the importance of modality selection and uniform diagnostic criteria when assessing for MAD.

MAD is also strongly associated with myxomatous degeneration of the mitral valve, particularly in individuals with advanced degenerative changes [13]. Prevalence rates of MAD in this subgroup range from 21.8% to 98%, with an average of 50.8% across two key studies [14,15]. A more nuanced classification distinguishes “true-MAD,” defined by its persistence in both systole and diastole, from “pseudo-MAD,” which is apparent only during systole [16]. A 2024 study demonstrated that true-MAD was present in 7% of MVP patients, while pseudo-MAD occurred in 37%. The latter was linked to more severe mitral regurgitation and greater myxomatous involvement [17].

Furthermore, MAD is frequently observed in syndromic MVP conditions, such as Marfan and Loeys–Dietz syndromes. The prevalence of MAD in patients with Marfan syndrome has been reported to be 34% and 46% in respective studies [18,19]. In Loeys–Dietz syndrome, MAD prevalence ranges from 34% to 37% [19].

## 3. Pathophysiology

The mitral annulus is a dynamic, D-shaped structure that undergoes continuous remodeling and deformation during the cardiac cycle. Regarding its structure, it consists of anterior and posterior segments, with the anterior segment characterized by its connection to the aortic valve, the aortomitral continuity, and the fibrous trigones [20]. In contrast, the posterior segment, which is less structurally stable, is more susceptible to abnormalities [3]. Mechanically, MAD alters the dynamic interaction between the mitral annulus and the basal left ventricular myocardium; this abnormal displacement during systole disrupts annular function and may impair valve coaptation [3,21].

MAD is thought to pose additional stress on the posterior mitral leaflet and chordal structures, particularly in the context of MVP or myxomatous degeneration [6]. This increased stress may lead to progressive remodeling of the mitral apparatus, exacerbating the severity of mitral regurgitation and contributing to ventricular remodeling [22].

Beyond its structural effects, MAD plays a significant role in electrical instability [23]. The abnormal motion and remodeling associated with MAD can create a substrate for arrhythmias [24]. These mechanical alterations may contribute to electrical instability, a topic explored further in the following sections.

The origin of MAD remains a subject of ongoing investigation [5]. Congenital MAD may result from abnormal embryonic development, whilst alternatively, acquired MAD may arise due to degenerative changes, left ventricular remodeling, or mechanical stress imposed by coexisting conditions, such as MVP or significant mitral regurgitation [6,25].

Emerging evidence suggests that MAD is not a static entity but a dynamic abnormality that varies throughout the cardiac cycle. This dynamic behavior may explain the challenges in diagnosing MAD and its variable clinical impact. The degree of disjunction and the extent of associated annular motion likely influence the severity of mitral regurgitation, arrhythmogenesis, and ventricular remodeling [26].

## 4. Diagnostic Methods

MAD is primarily identified using cardiovascular imaging, with TTE, TEE, cardiac computed tomography (CT), and CMR being the main diagnostic modalities. [27,28,29] Imaging typically reveals a systolic separation between the mitral valve annulus and the adjacent posterior wall of the left ventricle, most evident during systole [30]. However, the absence of fully established diagnostic guidelines and reference standards has contributed to variability in the detection and assessment of MAD [31]. Given the variability in terminology and imaging findings, Table 1 outlines key types of MAD, contrasting definitions such as true and pseudo-MAD and highlighting criteria that may indicate clinical significance.

Each imaging modality plays a distinct role in the evaluation of MAD. Echocardiography, particularly TTE, is the cornerstone for the anatomical and hemodynamic characterization of MAD [32]. TEE provides high resolution images, making it useful for more detailed evaluation [33]. On the other hand, CMR is considered the gold standard for detecting low-grade MAD, assessing its extent, and identifying coexisting myocardial fibrosis [27,31].

MAD most commonly affects the lateral and medial segments of the mitral annulus, specifically targeting the P1 and P2 scallops [28]. Consequently, these regions should receive special attention during imaging. Emerging evidence suggests that MAD may involve a larger portion of the mitral annulus than previously recognized, potentially affecting more than two-thirds of the structure [34]. Furthermore, the length of MAD varies along the annular circumference, often impacting the P1 and P3 regions while sparing P2 [8].

CMR, with its superior spatial resolution and advanced imaging protocols, allows for a more comprehensive assessment of the mitral valve and its associated abnormalities [35]. This modality not only enhances the identification of MAD but also enables precise evaluation of its relationship with coexisting conditions, such as myxomatous mitral valve degeneration and mitral regurgitation [27].

Given the strong association between MAD, myxomatous degeneration, and MVP, a thorough evaluation using advanced cardiac imaging is essential for patients with MVP and related symptoms, particularly when arrhythmias are suspected [31]. Comprehensive imaging facilitates risk stratification and guides clinical management by revealing subtle abnormalities that may predispose patients to arrhythmic events [11].

### 4.1. Transthoracic Echocardiography (TTE)

**Figure 1:**
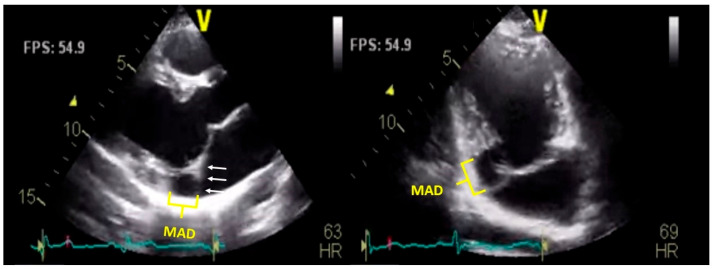
Transthoracic echocardiographic images demonstrating mitral annular disjunction (MAD). Parasternal long-axis view (**left**) and apical four-chamber view (**right**) reveal a distinct separation between the posterior mitral annulus (white arrows) and the basal inferolateral left ventricular myocardium (yellow brackets). The arrows highlight the disjunction plane in systole. MAD is visualized as a systolic detachment of the mitral annulus from the ventricular wall, most prominently affecting the posterior annulus.

TTE is one of the primary imaging modalities for the evaluation of MAD; it is widely available and non-invasive and provides real-time imaging of cardiac structures and function [36]. MAD is typically visualized in the parasternal long axis during systole, where a distinct separation is seen between the mitral valve annulus and the basal posterior LV wall (Figure 1) [12]. Although less commonly used for MAD assessment, the apical four-chamber and two-chamber views may also demonstrate this anomaly during the systolic phase [17].

However, TTE has limitations in detecting MAD, particularly in cases where image quality is compromised due to poor acoustic windows or when the mitral annulus is heavily calcified [37]. In addition, the sensitivity of TTE for MAD detection is lower compared to CT and CMR [31]. Despite these limitations, TTE remains the first-line tool for assessing MAD and the accompanying hemodynamic consequences, such as mitral regurgitation [38].

### 4.2. Transesophageal Echocardiography (TEE) and Three-Dimensional TEE (3D TEE)

TEE offers superior spatial resolution compared to TTE and is particularly useful for the detailed assessment of MAD [39]. During TEE imaging, MAD is best evaluated in the four-chamber view at 0° during the systolic phase and is defined as the separation between the point where the P2 scallop of the posterior mitral leaflet inserts into the atrial wall and the adjacent LV myocardium [33].

3D TEE further enhances the visualization of MAD by providing a detailed assessment of the mitral annulus. Unlike two-dimensional echocardiography, which provides segmented imaging, 3D TEE allows for a comprehensive visualization of the mitral valve from both the atrial and ventricular perspectives [40]. This capability significantly improves sensitivity for MAD detection and enables precise measurement of its extent [36].

Given its high spatial resolution, TEE, particularly with 3D reconstruction, is a preferred imaging modality in patients where TTE findings are inconclusive, when a poor acoustic window is present, or when a more detailed evaluation of the mitral valve apparatus is essential.

### 4.3. Cardiac Computed Tomography (CT)

Cardiac CT has emerged as a valuable tool in the assessment of MAD, particularly due to its high spatial resolution [31]. Cardiac CT enables detailed 3D visualization of the mitral valve and surrounding structures, aiding in the detection of annular abnormalities [41]. However, this diagnostic modality is accompanied by certain limitations; its primary drawback is its inability to provide dynamic, real-time assessment of the mitral valve motion, as it relies on retrospective electrocardiographic gating to reconstruct images [42]. Nevertheless, when performed with appropriate imaging planes, cardiac CT can offer an accurate anatomical assessment of MAD [11]. Cardiac CT is generally considered an adjunct to echocardiography and CMR rather than a first-line imaging tool for MAD evaluation.

### 4.4. Cardiovascular Magnetic Resonance (CMR)

**Figure 2:**
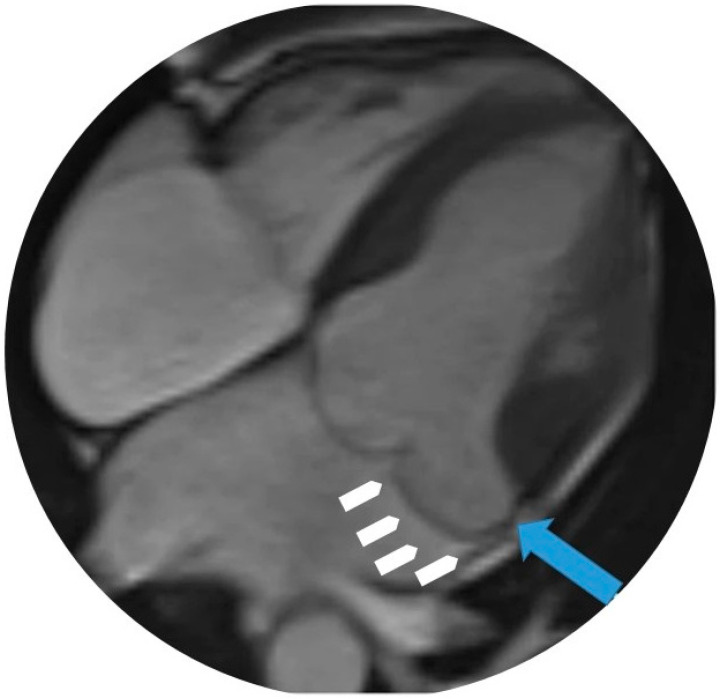
Cardiovascular magnetic resonance (CMR) image demonstrating mitral annular disjunction (MAD). In this long-axis cine frame, the blue arrow indicates the region of disjunction, seen as a systolic separation between the posterior mitral annulus (white arrows) and the basal inferolateral left ventricular myocardium. This finding is characteristic of MAD and best appreciated during systole on CMR cine imaging.

CMR is considered the gold standard for MAD assessment due to its ability to provide high-resolution, multi-planar imaging of the mitral annulus. Not only does it facilitate precise measurement of MAD, it also allows for comprehensive evaluation of myocardial structure and function, including LV volumes and myocardial fibrosis [27]. Studies have demonstrated that MAD is often associated with increased LV basal and mid-cavity diameters, as well as enlargement of the mitral annulus [8]. CMR can also identify myocardial fibrosis using late gadolinium enhancement (LGE), particularly in the regions surrounding the papillary muscles, which is more frequently observed in patients with MAD (Figure 2) [43].

The ability of CMR to detect subtle myocardial changes, particularly fibrosis, has important prognostic implications. Given the association between MAD, myocardial fibrosis, and an increased risk of arrhythmias, CMR plays a crucial role in risk stratification and guiding clinical management in patients with mitral valve prolapse (MVP) and related symptoms [44]. Additionally, CMR is highly reproducible, with the lowest intra-observer and inter-observer variability among the available imaging modalities, making it a preferred technique for precise and standardized evaluation of MAD [31].

### 4.5. Comparison of Imaging Modalities

The diagnostic yield of MAD varies depending on the imaging modality used [34]. Studies have demonstrated that CMR detects a higher prevalence of MAD compared to echocardiography [27]. The agreement in recognizing MAD is moderate between TTE and CMR and stronger between TEE and CMR [45]. When measuring MAD width, TTE and CMR demonstrate the highest correlation, while TTE shows moderate agreement with CMR [31].

TTE is associated with a lower MAD detection rate, which may be attributed to poor acoustic windows or artefacts caused by mitral annular calcifications [21]. Nonetheless, when assessing MAD length, no significant differences were identified between TTE, TEE, and CMR [11]. The reliability of MAD measurements is also influenced by intra-observer and inter-observer variability [31]. While all modalities exhibit some degree of measurement variability, CMR has demonstrated the highest agreement in repeated assessments, highlighting its role as the most reproducible imaging technique for MAD evaluation [27].

Overall, these findings emphasize the importance of standardizing MAD definition and measurement criteria in clinical practice to enhance diagnostic consistency and improve risk stratification of the affected patients.

## 5. Arrhythmias and Sudden Cardiac Death

In recent years, increasing attention has been given to the arrhythmogenic potential of MAD and its possible association with SCD. Although several observational studies have reported correlations between MAD and malignant arrhythmias, a definitive causal relationship has not yet been firmly established [5].

As previously discussed, MAD is recognized as a potential substrate for ventricular arrhythmias and SCD [46]. This interest stems from its frequent coexistence with MVP but also from evidence suggesting an independent arrhythmogenic role [23]. Several reports have highlighted the pathophysiological processes underpinning MAD-related arrhythmias; in these patients, paradoxical systolic expansion of the mitral annulus and adjacent left ventricular segments, typically involving the basal inferolateral wall and the papillary muscle insertion sites, leads to regional myocardial hypertrophy, subsequent cell death, and fibrotic replacement [47,48]. Chronic mechanical stress at the site of disjunction may further amplify these changes, establishing a substrate for reentrant ventricular arrhythmias [49].

Dejgaard et al. demonstrated a strong association between MAD and ventricular arrhythmias, including premature ventricular contractions (PVCs) and more severe manifestations such as non-sustained or sustained ventricular tachycardia (VT), even in the absence of MVP [47]. Supporting these findings, Yeerken et al. reported that 22% of patients with MAD did not exhibit MVP; among these, 38% experienced ventricular arrhythmias, reinforcing the notion that MAD can independently contribute to arrhythmogenic risk [48].

Symptomatically, palpitations are frequently reported among individuals with MAD, with rates as high as 71% in some cohorts [47]. The occurrence of malignant arrhythmias is rather concerning: up to 34% of patients may experience non-sustained or sustained VT, and approximately 9% may suffer cardiac arrest without any other apparent cause [11,50]. In the study by Carmo et al., atrial fibrillation (AF) was also reported, with 16% of patients exhibiting persistent AF and 11% having episodes of paroxysmal AF (PAF) [14]. However, the incidence of PAF was not significantly higher than in the general population, suggesting that ventricular rather than atrial arrhythmias are more closely linked to MAD.

Importantly, several reports suggest that the extent of annular disjunction may carry prognostic significance. A MAD distance exceeding 8.5 mm has been associated with a markedly increased risk of ventricular arrhythmias, offering 67% sensitivity and 83% specificity for predicting the presence of VT or frequent ventricular ectopy [12,14]. This highlights the importance of precise imaging and measurement in risk stratification.

Collectively, these findings underscore the need for heightened clinical vigilance in patients with MAD, especially those with a significant disjunction distance, ventricular arrhythmias, or symptoms such as palpitations or syncope. While the exact mechanisms and thresholds for intervention remain to be fully defined, MAD should be recognized as an important structural abnormality with potential arrhythmic consequences, even in the absence of MVP.

## 6. Therapeutic Implications

The identification of MAD has important therapeutic and prognostic implications, particularly in patients with coexisting MVP and ventricular arrhythmias. Increasingly recognized as a structural substrate for arrhythmogenesis, MAD warrants careful evaluation and may influence clinical decisions regarding monitoring, risk stratification, and therapeutic interventions [11].

In asymptomatic individuals with isolated MAD, normal ventricular function, and no documented arrhythmias or significant mitral regurgitation, a conservative management strategy with periodic clinical and imaging follow-up is generally appropriate [2]. However, the presence of symptoms, such as palpitations, unexplained syncope, or documented arrhythmias, should prompt more in-depth evaluation [11]. Ambulatory rhythm monitoring (e.g., Holter or long-term event recorder) and CMR are essential tools to detect arrhythmias, assess myocardial fibrosis, and evaluate left ventricular function [21,27].

For patients with confirmed ventricular arrhythmias and MAD, initial medical therapy typically includes beta-blockers or antiarrhythmic agents [26]. In cases of drug-refractory arrhythmias or when arrhythmias originate from identifiable foci, catheter ablation may be considered, although long-term success rates remain variable [51]. The co-presence of MAD and myocardial fibrosis on CMR, particularly involving the papillary muscles or basal inferolateral segments, may identify individuals at higher risk for SCD. In such cases, implantable cardioverter-defibrillator (ICD) therapy may be appropriate for primary or secondary prevention [11,27].

Surgical intervention is generally reserved for patients with significant MR and symptomatic MVP in whom MAD is also present [52]. Surgical mitral valve repair, by reattaching the mitral annulus to the ventricular myocardium, not only addresses the valvular lesion but may also reduce the burden of ventricular arrhythmias through elimination of the mechanical disjunction [20,53]. Although emerging data suggest a potential benefit of surgical correction in mitigating arrhythmic risk, these findings are based on retrospective cohorts and require confirmation in prospective studies [4].

**Figure 3:**
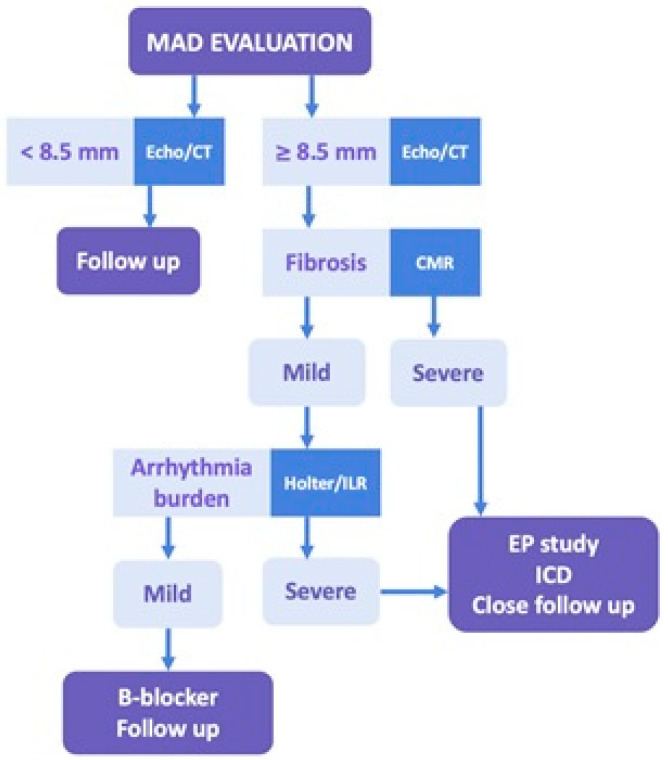
Proposed algorithm for arrhythmic risk stratification and management in patients with mitral annular disjunction (MAD). Abbreviations: MAD, mitral annular disjunction; CT, computed tomography; CMR, cardiovascular magnetic resonance; ILR, implantable loop recorder; EP, electrophysiologic; ICD, implantable cardioverter defibrillator.

Ultimately, the management of MAD should be individualized, involving a multidisciplinary team that integrates imaging, electrophysiologic assessment, and patient-reported symptoms. Shared decision-making, especially in patients with overlapping structural and arrhythmic risk factors, is essential to tailoring a treatment approach that balances arrhythmic risk mitigation with procedural considerations. A suggested management approach based on MAD severity, imaging findings, and arrhythmic burden is summarized in Figure 3.

## 7. Prognosis

The recognized association between MVP, MAD, and an elevated risk of ventricular arrhythmias highlights the need for meticulous arrhythmic surveillance in affected individuals. Regular rhythm monitoring, such as 24 h or extended Holter monitoring, is highly recommended to identify arrhythmias early and guide management decisions [35].

Surgical intervention on the mitral valve has the potential to correct MAD entirely; by reattaching the mitral annulus to the left ventricular myocardium, surgical mitral valve repair eliminates the disjunction gap and has been associated with a significant reduction in arrhythmic events [20]. However, this benefit has not yet been confirmed in randomized controlled trials, and surgical repair is currently reserved for patients with severe mitral regurgitation rather than as a standalone antiarrhythmic strategy [54]. Importantly, this anatomical correction is not achievable with current transcatheter approaches, such as transcatheter edge-to-edge repair.

Data regarding the impact of MAD on surgical outcomes remain mixed. While the presence of MAD does not appear to preclude successful mitral valve repair in patients with MVP and severe MR, other studies have suggested that unrecognized MAD may contribute to suboptimal surgical results and reduced long-term durability of valve repair [39,40]. Thus, preoperative imaging to assess for MAD is essential, and intraoperative strategies may need to account for this structural abnormality to optimize repair stability.

In specific subgroups, such as patients with Marfan syndrome, the coexistence of MAD may further compound arrhythmic risk. MAD in this population has been associated with an increased incidence of ventricular tachycardia, SCD, and a higher burden of PVCs [19]. Additionally, patients with MAD often demonstrate larger aortic root diameters [18]. Although the frequency of acute aortic syndromes does not appear to differ significantly between MFS patients with and without MAD, those with MAD require more frequent mitral valve interventions [18,19]. These findings support the need for intensified clinical follow-up, including rhythm surveillance and advanced imaging, such as CMR, particularly in connective tissue disease populations.

As previously noted, a disjunction distance greater than 8.5 mm is a significant prognostic marker for malignant ventricular arrhythmias, with good sensitivity and specificity [14]. Bennett et al. further demonstrated that the combination of an increased MAD length and LGE of the papillary muscles on CMR, particularly in the anterolateral wall, significantly correlates with arrhythmic risk [12]. These CMR findings may help stratify patients for more intensive monitoring or potential intervention.

Despite these insights, the effectiveness of antiarrhythmic therapy or surgical intervention in reducing the long-term risk of ventricular tachycardia remains uncertain. In the study by Essayagh et al., neither pharmacologic treatment nor mitral surgery significantly reduced the incidence of VT in patients with MAD [20]. Furthermore, while MAD is clearly associated with arrhythmias, its presence in patients with MVP has not been shown to confer an increased risk of all-cause mortality over a 10-year follow-up period [38].

## 8. Discussion

Although historically underrecognized, MAD has emerged as a clinically significant entity with important structural and electrophysiological implications [25]. Increasing evidence links MAD to MVP, malignant ventricular arrhythmias, and SCD, underscoring the need for heightened clinical awareness and structured diagnostic evaluation [55].

A critical gap identified through this review is the limited familiarity with MAD among clinicians; in many instances, MAD continues to be perceived as an incidental echocardiographic finding rather than a pathologic substrate with arrhythmogenic potential [56]. This under-recognition may result in missed opportunities for early detection, especially in patients presenting with unexplained palpitations, non-sustained ventricular tachycardia, or even cardiac arrest in the absence of overt structural heart disease. Given its potential association with SCD, timely identification of MAD may enable risk stratification and initiation of preventive strategies, including lifestyle counseling, pharmacologic management, or surgical referral when appropriate [47].

MAD often coexists with MVP, particularly in younger women and individuals with connective tissue disorders [19,57]. However, its occurrence is not limited to patients with leaflet abnormalities; studies have reported MAD in individuals without MVP, suggesting that its arrhythmogenic risk is not entirely dependent on valvular pathology [30]. This highlights the need to consider MAD in the broader differential diagnosis of arrhythmogenic mitral valve disease, beyond the context of MVP.

Accurate imaging plays a central role in the detection and evaluation of MAD. While CMR is considered the gold standard due to its high spatial resolution and ability to detect associated myocardial fibrosis, it is not universally accessible. In contrast, transthoracic echocardiography, when performed by experienced clinicians with a high index of suspicion, remains a valuable first-line tool [11]. Expanding training in echocardiographic techniques and promoting standardized imaging protocols are essential steps toward improving diagnostic accuracy in routine practice.

Despite growing recognition of its clinical importance, our understanding of MAD remains limited. Much of the available evidence stems from small, retrospective cohorts, restricting its generalizability. There is a pressing need for prospective, multicenter studies and randomized clinical trials to better define the prevalence of MAD, clarify its natural history, determine its independent prognostic impact, and evaluate therapeutic interventions. In parallel, the development of consensus-based diagnostic criteria and imaging standards will be essential for uniform recognition and management across clinical settings. In particular, future studies should aim to define reliable thresholds for arrhythmic risk stratification and clarify which patient subgroups may benefit from intensified surveillance, medical therapy, device implantation, or early surgical repair. Emerging electrophysiological mapping techniques may also help uncover arrhythmogenic substrates not visible with conventional imaging, offering a potential role in future risk stratification strategies.

In conclusion, while more evidence is needed to fully understand the natural history and prognostic significance of MAD, current data suggest that it may be associated with arrhythmic risk, particularly in patients with accompanying high-risk features such as myocardial fibrosis or bileaflet MVP. As such, MAD should not be regarded as a benign or incidental imaging finding. Increased clinical awareness, systematic imaging evaluation, and individualized monitoring are essential for optimizing care in this evolving area.

## Figures and Tables

**Table 1:** Summary of MAD definitions and clinical relevance. Abbreviations: MAD, mitral annular disjunction; LV, left ventricular; CMR, cardiovascular magnetic resonance; 3D, three-dimensional; TEE, transesophageal echocardiogram; TTE, transthoracic echocardiogram; SCD, sudden cardiac death; MR, mitral regurgitation.

Type of MAD	Definition	Imaging Appearance	Key Features	Potential Clinical Relevance
True MAD	Separation between posterior mitral annulus and LV myocardium in both systole and diastole	Persistent disjunction throughout the cardiac cycle (seen on CMR/TTE/TEE/3D TEE)	Structural abnormality, more stable feature	May be associated with higher arrhythmic risk
Pseudo-MAD	Apparent disjunction seen only during systole, due to leaflet motion or prolapse	Transient separation in systole only (seen on TTE/TEE)	Often related to mitral valve prolapse motion	Less clear association with arrhythmias
Significant MAD	MAD distance > 8.5 mm	Measured as linear distance between annulus and myocardium (systole)	Often involves basal inferolateral LV wall	Increased risk of ventricular arrhythmias and SCD
Subclinical MAD	Small MAD distance, typically asymptomatic	May be detected incidentally on imaging	No significant MR or arrhythmias	Clinical significance uncertain; often managed conservatively

## Data Availability

No new data were created or analyzed in this study. Data sharing is not applicable to this article.

## Abbreviations

The following abbreviations are used in this manuscript:

MAD	mitral annular disjunction
MVP	mitral valve prolapse
TTE	transthoracic echocardiography
TEE	transesophageal echocardiography
LVEF	left ventricular ejection fraction
CT	computed tomography
CMR	cardiovascular magnetic resonance
LV	left ventricular
3D-TEE	three-dimensional transesophageal echocardiography
LGE	late gadolinium enhancement
SCD	sudden cardiac death
PAF	paroxysmal atrial fibrillation
PVC	premature ventricular contractions
NSVT	non-sustained ventricular tachycardia
SVT	sustained ventricular tachycardia
MR	mitral regurgitation
MFS	Marfan syndrome
ICD	International Classification of Diseases
